# The development of suckling behavior of neonatal mice is regulated by birth

**DOI:** 10.1186/1756-6606-7-8

**Published:** 2014-02-10

**Authors:** Tomohisa Toda, Hiroshi Kawasaki

**Affiliations:** 1 Department of Biophysical Genetics, Graduate School of Medical Sciences, Kanazawa University, Takara-machi 13-1, Kanazawa, Ishikawa 920-8640, Japan; 2 Brain/Liver Interface Medicine Research Center, Kanazawa University, Kanazawa, Ishikawa 920-8640, Japan; 3 Department of Molecular and Systems Neurobiology, Graduate School of Medicine, The University of Tokyo, Tokyo 113-0033, Japan; 4 Department of Neurology, Graduate School of Medicine, The University of Tokyo, Tokyo 113-0033, Japan

**Keywords:** Birth, Sensory system, Development

## Abstract

**Background:**

Although the function of the sensory system rapidly develops soon after birth in newborn pups, little is known about the mechanisms triggering this functional development of the sensory system.

**Results:**

Here we show that the birth of pups plays an active role in the functional development of the sensory system. We first optimized the experimental procedure for suckling behavior using neonatal mouse pups. Using this procedure, we found that preterm birth selectively accelerated the development of suckling behavior in neonatal pups, but not that of motor performance, suggesting that the birth of pups regulates the functional development of the sensory system soon after birth.

**Conclusions:**

Taken together with our recent findings that birth itself regulates the initiation of sensory map formation in the somatosensory and visual systems, these results support the idea that the birth of pups actively regulates the anatomical and functional development of the sensory system.

## Background

The birth of pups is the most drastic environmental change in the entire life of mammals. Before birth, embryos are isolated in the uterus and are kept away from external sensory stimuli. Nutrients and oxygen are automatically supplied by their mothers. In contrast, soon after birth, newborn pups start to receive external sensory stimuli and need to process sensory information from the external world. Using sensory information, newborn pups need to search for nipples actively to obtain milk. Because the environment of pups before and after parturition changes drastically, it is plausible that the nervous system needs to change dynamically during this environmental transition. However, little has been uncovered about the roles of birth in the maturation of the brain.

The somatosensory system has been widely used for investigating the influence of extrinsic environmental and intrinsic genetic factors on sensory map formation and developmental plasticity, with profound implications for other circuits in the central nervous system [1-10]. Recently, we found that the birth of pups itself regulates the initiation of sensory map formation [11]. When preterm birth was induced by the progesterone receptor antagonist mifepristone or by ovariectomy, barrel formation in the primary somatosensory cortex was accelerated. Preterm birth also accelerated eye-specific segregation in the visual system. Thus, our findings shed light on a novel role of birth as an active trigger which initiates the formation of sensory maps [11].

Because the formation of anatomical sensory maps is regulated by birth, we hypothesized that the functional maturation of the sensory system is also regulated by birth. To test this hypothesis, we focused on suckling behavior, which is the behavior of neonatal pups to find nipples and obtain milk, because suckling behavior is observed soon after birth [12]. When a neonatal pup is placed in contact with a mother, the pup begins to search for a nipple, probes around it and subsequently holds it in the mouth [12]. Suckling behavior has been identified across many mammalian species, including rats, rabbits and non-human primates [12-15]. Because suckling behavior involves the somatosensory system [16,17], whose formation was found to be regulated by birth [11], we thought that suckling behavior is suitable for evaluating the functional maturation of the sensory system in neonatal pups.

Here, to examine the role of birth in the development of suckling behavior soon after birth, we determined an optimal experimental procedure for examining suckling behavior in mice. Then, we examined the effect of preterm birth on the development of suckling behavior. We found that the development of suckling behavior, but not that of motor performance, was accelerated by preterm birth. Thus, our results suggest that the birth of pups regulates not only the anatomical development of the sensory system, but also the functional maturation of the sensory system.

## Results

### The development of suckling behavior in mice

**Figure 1:**
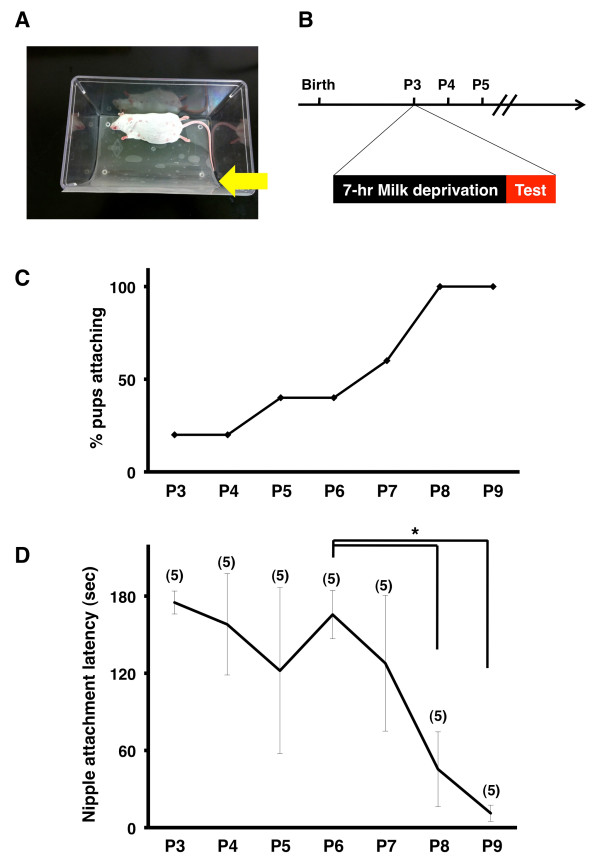
**The original test procedure for measuring nipple attachment latency. (A)** The experimental platform to test nipple attachment latency. The lactating dam was anesthetized and placed in a test cage. **(B)** The experimental procedure for measuring nipple attachment latency during development. Pups were separated from their mothers for 7 hours before the test. **(C)** The percentages of pups that were able to attach to a nipple within 180 sec. The percentage gradually increased after P4. **(D)** Latency between being placed in the cage and nipple attachment. Nipple attachment latency significantly decreased after P6 (*p < 0.05, Scheffe’s F test following Kruskal-Wallis test, mean ± SD).

Previously, suckling behavior has been mainly examined using rats [16,18]. However, because we recently found that birth regulates the initiation of sensory map formation using mouse pups [11], we first worked on finding an appropriate experimental procedure to investigate suckling behavior in mice. At the beginning, we tried the procedure previously used to investigate suckling behavior used in rats [16,18]. The lactating dam was anesthetized with pentobarbital to eliminate any movement, placed on her side in a test cage, and then tilted so that lines of nipples would be exposed to pups (Figure 1A) (for details, see Methods). Then, mouse pups at postnatal day 8 (P8) were separated from their mothers for 4 hours to avoid feeding, increasing their incentive to search for nipples. One of the pups was placed in close proximity to nipples, and the latency between the placement and attachment to the nipple (hereafter referred to as “nipple attachment latency”) was measured [18].

Unfortunately, however, a half of the pups failed to attach to nipples within a test period (180 sec), and therefore we were unable to measure nipple attachment latency for them (data not shown). We reasoned that the length of separation of pups from their mothers was not enough to fully motivate pups to search for nipples, presumably because some pups were not hungry enough. In fact, milk remaining in their stomachs was observed through the skin in some pups even after the 4-hour separation (data not shown). Consistently, it was reported that it took more than 4 hours for neonatal pups to digest milk [18]. To increase their incentive to search for nipples, we increased the time of the separation. As expected, after separation for 7 hours, all of pups searched for nipples more actively and eventually attached to nipples within 180 sec at P8 (data not shown). We therefore decided to use a separation time of 7 hours to examine nipple attachment latency in the following experiments (Figure 1B).

We then examined nipple attachment latency during development. As reported previously using rats [18], the percentages of pups who succeeded to attach to nipples within 180 sec gradually increased after birth in mice (Figure 1C). Nipple attachment latency significantly decreased between P6 and P9 (P6, 165.6 ± 18.8 sec; P8, 32.5 ± 29.0 sec; P9, 7.0 ± 6.3 sec; P6 vs P8, p < 0.05; P6 vs P9, p < 0.05; Scheffe’s F test following Kruskal-Wallis test) (Figure 1D and Additional file 1: Movie S1). This result was consistent with the idea that the ability of pups to find nipples markedly improves during this period.

**Figure 2:**
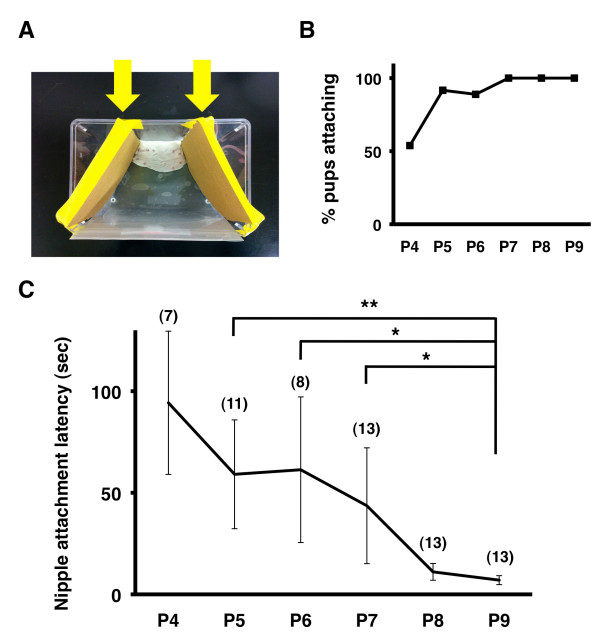
**The modified test procedure for measuring nipple attachment latency. (A)** The modified experimental platform. Two walls (arrows) were added to prevent pups from moving away from the nipples. **(B)** The percentages of pups that were able to attach a nipple within 180 sec. The percentage exceeded 85% after P5. **(C)** Latency between being placed in the cage and nipple attachment. Nipple attachment latency significantly decreased between P5 and P9 (*p < 0.05, **p < 0.01, Scheffe’s F test following Kruskal-Wallis test, mean ± SD).

However, we noticed that younger pups often failed to find nipples because they completely lost their way and went to the lactating dam’s head or tail, resulting in the failure to find nipples (Figure 1C). This seemed to prevent accurate measurement of nipple attachment latency, and therefore it was desirable that most pups eventually find nipples within 180 sec. In order to ensure that pups did not lose their way to the nipples, two additional walls were placed in the test cage (Figure 2A, arrows), preventing pups from going to the dam’s head or tail. One wall was placed rostral to the pectoral nipples, and the other one was placed caudal to the inguinal nipples (Figure 2A). These walls efficiently increased the probability that pups were able to find nipples within 180 sec (Figure 2B). More than 85% of pups at or older than P5 successfully attached to nipples within 180 sec (Figure 2B), and we therefore used these walls in the subsequent experiments. It should be noted that even when using the walls, a few pups failed to reach nipples within 180 sec. In order to precisely measure changes in nipple attachment latency as pups developed, we limited our measurements to pups who were able to attach to a nipple within 180 sec. Using this procedure, with which nipple attachment latency seemed more precisely to reflect the ability of pups to find nipples, we examined the developmental changes in nipple attachment latency.

We found that nipple attachment latency indeed significantly decreased between P5 and P9 (P5, 59.1 ± 26.8 sec; P6, 47.0 ± 35.8 sec; P7, 43.6 ± 28.5 sec; P8, 11.1 ± 4.1 sec; P9, 7.0 ± 2.2 sec; P5 vs P9, p < 0.01; P6 vs P9, p < 0.05; P7 vs P9, p < 0.05; Scheffe’s F test following Kruskal-Wallis test) (Figure 2C). This result suggests that the ability of pups to find nipples significantly improves during this period.

### Suckling behavior requires somatosensory inputs from the infraorbital nerve

**Figure 3:**
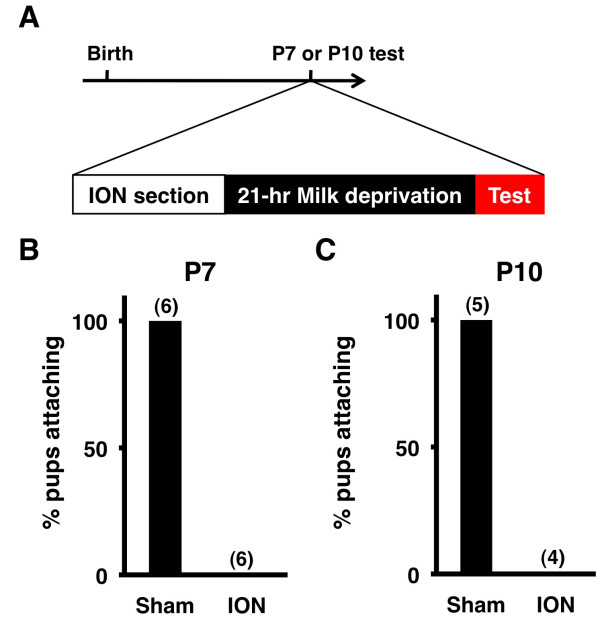
**The inhibition of somatosensory inputs prevents pups from finding nipples. (A)** The experimental procedure. After ION section was performed, pups were separated from their mothers for 21 hours, and then nipple attachment latencies were examined. **(B and C)** The percentage of pups that were able to attach to a nipple within 180 sec. ION-sectioning at P7 and P10 resulted in pups being unable to find and attach to nipples within 180 sec, while sham-operation did not apparently affect suckling behavior.

Since previous studies using animals other than mice demonstrated the importance of somatosensory inputs from the snout in finding nipples [15,16,19], we examined whether somatosensory inputs from the snout are also important for the reduction of nipple attachment latency in developing mice. To eliminate somatosensory inputs, the infraorbital nerve (ION) was cut (Figure 3A), and the operated pups were separated from their mothers for 7 hours or 21 hours to recover from the operation and increase their incentive to find nipples.

At the beginning, to determine how long it takes for operated pups to recover from the operation, we compared nipple attachment latency between sham-operated pups and non-treated control pups at P10. We found that the nipple attachment latency of sham-operated pups was larger than that of non-treated pups 7 hours after the operation, suggesting that 7 hours are not enough for operated pups to recover from the operation (non-treated pups 8.8 ± 2.5 sec, sham-operated pups 38.5 ± 21.4 sec, p < 0.05, Welch’s *t*-test). In contrast, when recovery was extended to 21 hours, we did not find any significant difference in nipple attachment latency between non-treated pups and sham-operated pups, suggesting that 21 hours are enough for operated pups to recover (non-treated pups 9.8 ± 5.4 sec, sham-operated pups 12.7 ± 4.7 sec, p = 0.42, Student’s *t*-test). Thus, we decided to allow operated pups to recover for 21 hours before the suckling behavior test. We then performed ION section, allowed the operated pups to recover for 21 hours, and examined whether pups attached to nipples within 180 sec. Interestingly, we found that ION section completely prevented all pups from finding nipples at both P7 and P10 even though they actively rooted into their mothers’ ventrum (Figure 3B, 3C and Additional file 2: Movie S2). This result suggests that suckling behavior requires somatosensory inputs from the snout.

Although it seemed likely that the lack of somatosensory inputs led to the effect of ION section, it also seemed possible that pain caused by ION section resulted in the effect. We believe the latter is unlikely because of the following reasons. First, the activity of ION-sectioned pups was indistinguishable from that of non-treated pups. Even though ION-sectioned pups were unable to attach to nipples, they actively rooted into their mothers’ ventrum and probed around nipples. If the pain of ION section were severe, their behavior would be quite different from that of non-treated pups. Second, the body weight of sham-operated pups increased similarly to that of non-treated pups. We performed sham operation at P7 and measured the body weight one day after recovery. We did not find a significant difference between sham-operated pups and non-treated pups (non-treated pups 4.7 ± 0.22 g, sham-operated pups 4.87 ± 0.32 g, p > 0.5, unpaired Student’s *t*-test), suggesting that pain derived from our surgical procedure did not affect suckling behavior. Taken together, these results suggest that somatosensory inputs are indeed important for suckling behavior.

### The birth of pups regulates the development of suckling behavior

**Figure 4:**
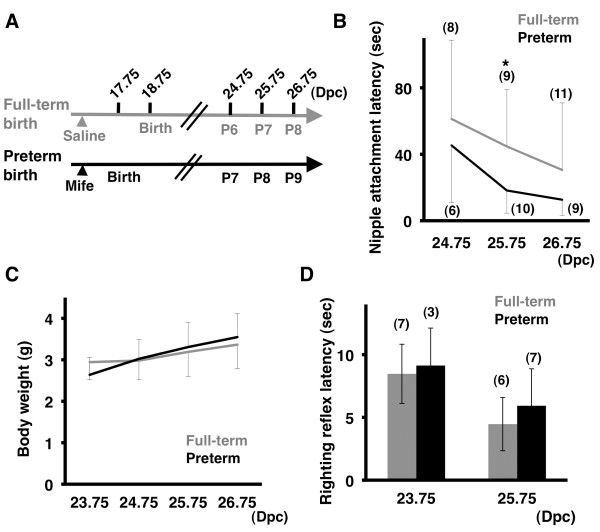
**Birth regulates the development of suckling behavior. (A)** The experimental procedure to induce preterm birth using mifepristone. Mifepristone (Mife) or saline was injected between 21:30 and 22:30 at 16 dpc. **(B)** Nipple attachment latency was reduced markedly earlier in preterm pups than in full-term pups. At 25.75 dpc, nipple attachment latency was significantly smaller in preterm pup than in full-term pups (*p < 0.05, Mann–Whitney U test, mean ± SD). **(C)** Body weight was not affected by preterm birth (p > 0.4, Student’s *t*-test, mean ± SD). **(D)** The righting reflex latency was not affected by preterm birth (p > 0.4, Student’s *t*-test, mean ± SD).

Recently, we reported that the birth of pups actively regulates barrel formation in the somatosensory cortex and eye-specific segregation in the visual system of neonatal mice [11]. Our findings highlighted the active roles of birth in neural circuit formation in neonatal pups. We therefore hypothesized that the birth of pups also regulates the development of suckling behavior. To test this hypothesis, we compared nipple attachment latency in preterm pups and that in full-term pups during development. As we described previously [11], we induced preterm birth by injecting the progesterone receptor antagonist mifepristone at 16.75 days post coitus (dpc) (hereafter we use dpc instead of postnatal days to indicate specific time points clearly, including those after birth, because postnatal days in preterm pups and those in full-term pups correspond to different developmental time points, as shown in Figure 4A. For example, postnatal day 6.0 (P6) corresponds to 23.75 dpc in preterm pups and 24.75 dpc in full-term pups).

As we expected, we found that preterm birth accelerated the reduction of nipple attachment latency (Figure 4B). The nipple attachment latency of preterm pups was significantly shorter than that of full-term pups at 25.75 dpc (full-term, 44.7 ± 34.5 sec; preterm, 18.2 ± 13.7 sec; p < 0.05, Mann–Whitney U test) (Figure 4B). These results suggest that birth regulates the development of suckling behavior.

It seemed possible that birth selectively regulates the development of suckling behavior. Conversely, it seemed also possible that birth regulates overall development of pups, and as a result of an indirect effect of preterm birth, suckling behavior was also affected by preterm birth. To distinguish between these possibilities, we measured the body weight of pups because the body weight of pups increases gradually after birth and because it seemed likely that the body weight is related to suckling behavior [18]. We did not find significant differences of the body weight between preterm pups and full-term pups at all ages we examined (Figure 4C). This is consistent with our previous observation showing that the increase in the body weight between 23.75 dpc and 24.75 dpc is not affected by preterm birth [11]. These results suggest that the overall development of pups is irrelevant to when pups are born, and it seems unlikely that the acceleration of the development of suckling behavior in preterm pups is an outcome of an indirect effect of accelerated overall development of pups.

Although we have shown that suckling behavior requires somatosensory inputs, the reduction of nipple attachment latency during development could be due to the improvement of motor performance of neonatal pups. To test this, we examined the latency of the righting reflex. Mouse pups often fall on their back and then right themselves onto all four feet, and we measured how much time it takes to right themselves. Consistent with previous studies [20,21], the righting reflex latency decreased between 23.75 dpc and 25.75 dpc in full-term pups (Figure 4D), indicating that motor performance of pups develops during this period. Interestingly, we found no significant difference in the righting reflex latency between full-term pups and preterm pups (Figure 4D), suggesting that the improvement of motor performance after birth is irrelevant to when pups are born. Thus, these results support the idea that preterm birth accelerates the development of suckling behavior by improving sensory functions of neonatal pups.

## Discussion

Here we developed an experimental procedure for examining suckling behavior using neonatal mice. Our modifications such as the increased length of the separation of pups from their mothers and the addition of walls in the test cage resulted in reproducible results for nipple attachment latency. Using this improved procedure, we have demonstrated that nipple attachment latency significantly decreased after birth in mice. Furthermore we uncovered that somatosensory inputs are required for suckling behavior in mice. Preterm birth accelerated the reduction of nipple attachment latency during development, while it did not affect that of righting reflex latency, suggesting that the birth of pups predominantly affects sensory functions rather than motor functions. Taken together with our previous report that preterm birth accelerates somatosensory map formation in the barrel cortex [11], our findings suggest that birth regulates not only anatomical somatosensory map formation but also the maturation of sensory functions during development.

We showed that ION section strongly inhibited suckling behavior. This result suggests that somatosensory inputs from the snout are essential for suckling behavior during development. Interestingly, when suckling behavior is developed soon after birth, somatosensory circuits, including whisker-related patterns of thalamocortical axons (TCAs), barrels and intracortical circuits such as barrel nets, are formed in the somatosensory cortex [2,6,7,10,22-27]. This coincidence implies that the maturation of somatosensory circuits contributes to the development of suckling behavior.

The birth of pups is one of the most drastic environmental changes in the entire life of mammals. It seems likely that newborn pups have to adapt to this drastic environmental change and have to start using the sensory system to survive soon after birth. Interestingly, we demonstrated that the birth of pups selectively regulates the initiation of sensory map formation in the somatosensory and visual systems during development [11]. Although these results uncovered a novel role of birth in the anatomical development of the brain, the importance of birth in functional maturation still remained to be elucidated. In this report, we have shown that the birth of pups regulates the development of suckling behavior. Taken together with our previous report [11], it seems reasonable to conclude that the birth of pups plays crucial roles in brain maturation anatomically and functionally.

Previously, experimental procedures for measuring nipple attachment latency were designed for use with animals other than mice [13-15,18]. We showed that our improved procedure was useful for elucidating developmental changes in nipple attachment latency day by day using mice (Figure 2) and for examining the effect of preterm birth on nipple attachment latency (Figure 4). Thus, our procedure should be useful for investigating the sensory function of mutant mice that may have sensory deficits. Although a number of mutant mice have abnormalities in their sensory map formation [6,23,28,29], little is known about their behavioral abnormalities in neonatal mice. It would be intriguing to compare the anatomical deficits and behavioral abnormalities of various mutant mice using our procedure.

## Conclusions

In summary, we demonstrated that the birth of pups regulates brain development not only anatomically but also functionally. We successfully found that preterm birth significantly accelerated the development of suckling behavior, suggesting that birth indeed regulates functional brain development. Interestingly, the development of motor performance was independent of birth, suggesting that birth regulates the development of the sensory system related to suckling behavior.

## Methods

### Animals

All procedures were performed in accordance with a protocol approved by the University of Tokyo Animal Care Committee and with a protocol approved by Kanazawa University Animal Care Committee. ICR mice (SLC, Japan) were reared under the normal 12 h light–dark cycle. The day of insemination was designated as 0 dpc. Gestation days were counted in 6-hour increments (ex. in this paper, 18.75 dpc indicates the period between 15:00 and 21:00 at 18 dpc). To ensure data reflected the precise time of delivery, pregnant mice were monitored using digital video recorders as described previously [11].

### Suckling behavior test

Our procedure for examining the suckling behavior of neonatal mice was made by modifying the procedure used for neonatal rats [18]. A lactating dam was anesthetized with pentobarbital to eliminate any movement and to block milk letdown. A sheet of plastic paper was placed in a test cage as described previously (Figure 1A, arrow) [18] because the plastic paper helped pups to keep in contact with the dam’s ventrum. The anesthetized dam was placed on her side in the test cage and tilted at an angle of 45° so that both lines of nipples would be exposed to pups. In addition, two walls, one rostral to the pectoral nipples and the other caudal to the inguinal nipples, were placed in the cage to prevent pups from moving far from the nipples (Figure 2A, arrows).

Before starting the test, pups were separated from their mothers and kept on a warm plate with home cage tips for the indicated time period (4, 7 or 21 hours). At the beginning of the suckling behavior test, the pups were placed in close proximity to the nipples on the ventrum of the anesthetized dam without contacting the nipples, and the time until the pups attached to nipples was measured. All tests were videotaped, and the latency between being placed in the cage and attaching to a nipple was recorded for each pup. A few pups failed to reach nipples within 180 sec, and in such cases, we excluded those pups from our analyses.

It should be noted that nipple attachment latency seemed to be affected by maturation of the dam’s nipples. It seemed that nipple attachment latency tended to be longer when mothers had small nipples. Therefore, in order to obtain stable and reproducible results for nipple attachment latency, when we compared nipple attachment latency between preterm and full-term pups, we used the same anesthetized mother for different littermates rather than using their biological mothers.

### Righting reflex test

To examine the development of the pups’ motor performance, a righting reflex test was performed. Mouse pups often fall on their back and then right themselves onto all four feet, and we measured how much time it took to right themselves onto all four feet as described previously [20,21].

### Infraorbital nerve (ION) transection

ION transection was conducted as described previously [2]. Pups at P7 or P10 were anesthetized with isoflurane, and the ION was exposed by making a vertical slit just behind the whisker pad. The ION was identified under a dissecting microscope and was cut with a pair of iridectomy scissors. The resection stump was subjected to electrical cautery using a cautery device to prevent nerve regeneration. The wound was closed with Vetbond (3 M), and ofloxacin ointment was applied to the wound to prevent infection. The IONs on both sides were cut. Sham operation was performed similarly, but the IONs were left intact. After the operation, the pups were revived and kept on a warm plate without their mothers for 7 hours or 21 hours, after which they were subjected to the suckling behavior test.

### Induction of preterm birth

To induce preterm birth, mifepristone treatment was carried out as described previously with modifications [11,30]. The day of insemination was designated as 0 dpc. Gestation days were counted in 6-hour increments (ex. in this paper, 18.75 dpc indicates the period between 15:00 and 21:00 at 18 dpc). To ensure data reflected the precise time of delivery, pregnant mice were monitored using digital video recorders as described previously [11].

### Statistical analyses

Statistical analyses were performed using Statcel2 software (OMS Publishing, Japan) and R software. To assess statistical significance, we first performed the Kolmogorov-Smirnov test. P values were determined by an unpaired Student’s *t*-test, Welch’s *t*-test, the Mann–Whitney U test and the Kruskal-Wallis test with the Scheffe’s F test. “n” means the number of animals.

## Abbreviations

ION: Infraorbital nerve.

## Competing interests

The authors declare that they have no competing interest.

## Authors’ contributions

TT and HK designed the experiments; TT performed the experiments; TT analyzed the data; TT and HK wrote the paper. Both authors read and approved the final manuscript.

## Supplementary Material

Additional file 1 **Movie S1.** Suckling behavior in mouse pups at P6 and P9. Click here for file

Additional file 2 **Movie S2.** Suckling behavior in sham-operated and ION-sectioned pups. Click here for file
